# Secondary Syphilis

**Published:** 1854-02

**Authors:** J. Grafton

**Affiliations:** Janesville, Wis.


### *Secondary Syphilis; death from falling in of the Epiglottis.*

BY J. GRAFTON, M. D. JANESVILLE, WIS.

A. H. aged 30. Sanguine temperament, has been a free liver, but not intemperate, 3 or 4 glasses of ale with one of Brandy constituting his daily modcum. Had uniformily enjoyed good health until the period of contracting Syphilis 12 years ago, when he became the subject of a severe indurated Chancre, cured by mercury and the topical application of black wash, producing severe salivation.

Three months afterwards, secondary, symptoms of vescular character appeared, for which he was again salivated, Iodide of Potassium and sarza having vailed to benefit him. This eruptive attack was *severe* and protracted, followed soon afterwards by tertiary symptoms which he says were chiefly of a rheumatic character. His hair fell off, his general health failing him, leaving him a martyr to Rheumatism. The long bones, clavicle, and more especially the costœ, bear prominent marks of long continued syphilitic periostitis being extremely nodular. With a view of thoroughly eradicating the syphilitic poison,has on several occasions taken "*courses* of mercury," has literally lived upon Iodide of Potassium, Arsenic, and Sarsaparilla,purchasing the former "by the pound." He has also taken liberally iron and nitric acid.

Apr. 15th, 1853. He contracted chancre upon the upper part of the glans,which healed upon the use of Black wash and an occasional blue pill; has also taken as usual the Iod. Potass., Liq., Arsenic and Sarza.

May, 26th. Contracted a severe cold, and soon afterwards called upon my friend Dr. Lewis for advice, stating that he had experienced a severe chill, that his bones ached. His throat was sore and his breathing much embarrassed. For these symtoms the Dr., prescribed Pil. Hydrary, Gr. iv. bis die, and a mixture of chlorate of Potash.

May 27th. Unable to speak, except in a hoarse whisper—throat more sore, revealing on inspection a very angry appearance, especially on the inferior surface and sides of the velum. The vivid redness of yesterday has given place to large aphthous patches of an ashy hue, with extensive and increasing œdemia, breathing much more difficult, with considerable fever. Dr. L. applied solid nitrate of silver to the patches on the velum, repeated the blue pill and chlorate mixture, with sinapisms to the throat.

May 28th. Dr. Lewis invited me to visit him, intimating his suspicion that the symptoms now much aggravated, were dependent, in some degree, at least,upon syphilitic poison. At 7 P. M. found him in bed,bathed in perspiration,complaining of much difficulty of breathing, respiration 35, pulse 136, small and feeble, mouth wide open, face red and swollen. His dyspnœa *uniform,* not paroxysmal; has frequent cough, which he thinks is occasioned by accumulation of a viscid saliva, about the back of the fauces; cough remarkably hoarse; throat "uncomfortable," but not sore; swallows liquids and solids without much pain; does not complain of pressure over the larynx, ortrachea. If there is tenderness anywhere, it is immediately above the hyoid bone, when pressed backwards and upwards, towards the base of the tongue. Has never expectorated bloody or purulent matter, had no previous symptoms of disease of the larnygeal or tracheal cartilages; his tonsils have never suffered; the only affection of the throat he remembers, has been occasional excoriation in bad weather. Within the last four days, he has choked a little while drinking.

His present symptoms, although inflammatory, and of laryngeal location, differ much in their character from any other case of syphilitic or idiopathic laryngitis I have ever seen. He has none of that terrible restlessness consequent upon the fear of immediate suffocation; there is no evidence of want of proper oxygenation of the blood. He is not drowsy; his lips are not blue, nor is his countenance livid. His inspiration is comparatively noiseless, without stridulous sound; *hurried,* rather than protracted. His cough is hoarse and not brassy. He does not grasp for breath or demand pure air, preferring to keep his door and window shut, although his apartment is very small, and the weather extremely warm. In consequence, the air is exceedingly offensive, tainted with the effluvia from the various discharges of the numerous sores about his person, and the not less intolerable odor of his syphilitic perspiration. His objection to ventilation is the dread of contracting pain in his bones, to guard against which, he is now clothed in four wrappings of flannel. On examining his throat, I was surprised at the extreme contraction of the isthmus faucium, the passage into the pharynx being almost obliterated; so much so that when afterwards applying a solution of Nitr. Ar., I was compelled to reduce the size of the sponge on my gargeal probang, to one-third of its diameter. The appearance of the passage is that of a narrow slit, perceptible only on depression of the tongue, upon which the soft palate lies. The uvula is completely hidden;when lifted up,extremely livid and ædematous. The arches of the palate and triangular recess between its pillars, aro quite lost in the general tumefaction in which the mucous membrane at the base of the tongue participates. Depression of this organ is effected with difficulty, causes pain and violent expulsive coughing, so much so as to render it impossible to catch a view of the deeper seated parts. On passing my finger over the edges of the rima, I found it less œdematous than I expected although considerably swollen and cushion-like.

The epiglottis was much more timid, semi-erect, projecting backward from the base of the tongue. The mucous membrane forming at its base the gloss o-epiglottidean folds, being thrown upwards, so as nearly to obliterate the normal interval between its anterior surface and the dorsum of the tongue. The general appearance of the parts resembles that seen in a person severely scalded by drinking hot water, differing chiefly in the color of the membrane, which is here of an ashy hue and evidently about to slough. At present the sloughing process is most apparent on the velume, being confined chiefly to the epithelial lager. On the sides of the fauces, and base of the tongue, the sloughs are of a more serious character and involve the whole thickness of the membrane. These sloughs are bounded anteriorly by a well defined line of demarcation. On passing the finger over the velum, on its anterior surface, I discovered the walls of an ulcer, with a soft spot in the centre, as if about to break through, into the mouth; evidently indicating the existance of an ulcer on its posterior surface. The fœtor of his breath, together with that from the nose, was most intolerable. He has a large syphilitic tubercle on the tip of his nose, another at the junction of the left ala with the cheek, both in a state of ulceration, covered over with heavy dark brown crusts one-fourth of an inch in thickness, which when detached, reveal foul ragged excavating ulcers. He attributes these sores which are undoubtedly syphilitic, to the effect of severe cold, having frozen his nose about twelve months ago. They commenced some months back, in the form of little hard tubercles the size of a pea, stationary for a time, then ulcerating on their surface, crusting and re-crusting over as at present, steadily increasing in breadth and depth. The mucous membrane lining the nasal passages, is one mass of ulceration, extending backward to the pharynx, involving the turbinated and ethmoid bones. A foul sanious discharge constantly oozes from his nostrils, which are filled with crusts. The sclero tic coat of the eye, is inflamed; around the corner, about a line from its circumference, is a well defined zone of dusky red vessels, situated *beneath* the conjunctiva. The Iris has lost its circular outline, and is of a dirty greenish hue,

(no effused lymph); has no pain except at night, when the eye feels a "little uneasy." On his face, especially about the cheeks, are numerous cicatrices of former eruptive attacks, interspresed on the breast and arms with numerous copper colored stains of more recent origin. On his hands are several sores, covered with greenish yellow scales, on his breast are two larger vesicles in process of filling. He looks haggard; is feeble and dispirited. His sleep and appetite have failed him for some weeks. He is probably on the eve of another attack of eruptive syphilis. There can be but little doubt as to the specific nature of the disease; how then shall we treat him ? Have we any immediate and reliable remedy to combat the specific inflammation, now going on within the fauces, which will not aggravate that tendency to sloughing, already so imminent ? His embarrassed respiration must be immediately relieved; a slight increase of the existing tumidity will altogether prevent respiration and leave no other resource than tracheotomy. His former attacks of secondary syphilis were cured only by mercury. His present symptoms have developed themselves in the very teeth of those remedies usually deemed specifics; viz : iodide of potash, sarsaparilla and arsenic. Shall we trust to mercury ? Apart from that knowledge which theory and experience have taught us respecting its pernicious influence in all cases where sloughing is threatened, more especially where it already exists, in a neighborhood too of such vital relations, the slightest extension of which may hurry him out of existence in a moment, by shutting down the very gate of life, or driving him yet more rapidly into eternity by the rending asunder of those fragile walls whose disruption would drown his very soul in blood, we have yet additional reason for caution in the administration of this remedy.

The constitutional powers of our patient are well nigh broken down. Syphilitic disease, and its not less terrible antidote, have long been struggling with life for the mastery. He is the very impersonation of spanœmia; pale and exsanguine, with flabby muscles; his nervous system shattered by debility of his own begetting. What have we to expect but sloughing, if we reduce him more, when in addition we remember that for days past, he has been living in a very "forcing house" of gangrene?—shut up in a foul aad pestilential atmosphere breathing in again those putrid exhalations which nature had cast off in her loathing.

Influenced by these considerations, we concluded to discontinue the use of blue pill and substitute for this evening, the topical use of cinnabar by inhalations, ordering the following detergent gargle to cleanse out and check that viscid secretion which must from its very adhesiveness materially interfere with the small column of air passing into the larynx; a fact convincingly attested by the frothiness of the sputa, now hanging to the fauces. ℞. Hydrargyri Bichloride gr 1. Ammoniæ Hydrochl ʒi; Aqua Pluv. ℥ viij. Mix. Use as a gargle. To continue his chlorate of potash, and take 10 gr. of Dover's powders. Door and window to be left open, protecting him from the draught by a curtain; to have strong beef tea in addition to his arrow root.

May, 29. Has inhaled 10 grains of cinnabar and taken medicine ordered. Feels more comfortable; much relieved in his breathing. The viscid secretion is now removed from his mouth; is persuaded that his throat is better; had a good night; does not perspire so much. Pulse 130, respiration 26. The throat is improved in appearance. Tumefaction somewhat diminished; slough on the surface of the velum, separating kindly, and does not appear to extend deeper than the epithelial layer. Coughs less; has taken some nourishment. Ordered continuance of the medicines, and to resume use of iodide of potash and sars, applied a solution of arg. nitrate, in nitric acid and water to sloughing surface. In evening improved in every respect; cheerful; repeat remedies, and take a little bottled Ale, with brandy in his arrow root.

30th, Morning; breathing tranquil; feels weak; had a severe fit of coughing in the night. Threw up a triangular piece of mucous membrane, an inch and a half wide at its base, one half an inch at its apex, and about two inches in length. Was nearly choked by it; had to pull it away. This slough comprises the whole thickness of the mucous membrane, its under surface being evidently of a fibro-vascular charater; detachment *not* attended with hemorrhage. On examination found the velum assuming a more healthy condition, a process of reparation having evidently begun. The uvula is still œdematous livid and gangrenous. Fauces much swollen; on forcibly depressing the tongue, I caught for the first time, a momentary view of the epiglottis, which appeared at least thrice as thick as it should be, and livid. The mucous membrane detached this morning, appears to have been that portion lining the triangular recess between the palatine arches; the denuded surface looks clean and healthy. The slough at the base of the tongue shows evidence of separation; the line of demarcation is more swollen and livid. I can raise the anterior edge with the handle of a spoon. His breath is again more fœtid: ordered the nitrate to be applied; to discontinue cinnabar, and use the other medicine, with more nourishment. Respiration 20. Pulse 120.

8 P. M. Visited him again; looks paler. "has a feeling of sinking in his chest," voice more hoarse; does not breathe as well as in the morning, chokes more while drinking. No appreciable change in the condition of the throat. No pain in chest; respiration 25; pulse 133, very feeble. Ordered nourishment during the night, at stated intervals; to take one half a grain of morphia; "says he has several times coughed up little shreds of membrane and some pus.

31st. His attendant called to announce his death, which must have been instantaneous, and without a struggle, as he died withot the knowledge of his attendant who slept on the floor by his side. At 10 o'clock, P. M., he called for Ale; said he felt easy, and likely to sleep; wanted rest more than anything. At 12 o'clock he was heard to cough and clear his throat as usual; at 6 o'clock he was found dead on his back, without any disturbance of the clothes, or any sign by which to indicate that he had any warning of his decease. Being pressed to give an opinion respecting the cause of his death, I expressed my belief, that the sloughing had extended to the ligaments of the arytenoid cartilage,' allowing them to fall in, and so suffocate him; or what was more probable, that the slough at the base of the tongue had given way, and allowed the epiglottis to fall over and close the rima. This I expressed as a conjecture, and requested permission to make a post mortem examination.

Permission was given only to examine the throat. Autopsy. The parts having been carefully taken out and exposed, the mucous membrane lining the posterior wall of the pharynx, was found intensely red, and in a pulpy condition, not extending farther than the commencement of the œsophagus, which when slit open, presented a perfectly healthy appearance, the mucous membrane being pale and bloodless. The sides of the pharynx were coated with a greasy albuminous exudation, the mucous membrane in places altogether destroyed. The anterior inferior wall of the velum evidently showing traces of reparation as far as the uvula, thee pithelial lining being in part removed. On its superior or nasal surface the half of an excavated ulcer, the size of a large bean; its surface being covered with a foul green slough. Uvula gangrenous throughout. Mucous membrane forming sides of the pharynx, in a sloughing condition as far as the lower border of the middle constriction.

On raising the uvula and distending the pharynx, the cause of death was at once apparent. The triangular opening into Larynx was com pletely closed by the falling in of the epiglottis, occasioned by sloughing and subsequent se paration of the mucous ligaments connecting it with the base of the tongue, a portion of which was drawn tightly through the rima, probably by forced inspiration and retained firmly fixing the epiglottis in its abnormal position. The cellular tissué and glands at the base of the tongue were one mass of putridity. The mucous membrane ef the superior aperture of the larynx forming the aryteno-epiglottidean folds on either side, was in a state of *ulceration*, its surface rough and granular, deprived of its epithelium, coated with a thick purulent secretion, extending into the interior of the larynx, the mucous membrane of which was found to be in the same condition, as low down as the centre of the cricoid cartilage, the Laryngeal pouches filled with pus; the superior or false vocal chords partially destroyed; the mucous lining of the inferior or true vocal chord granular and coated with pus; at the base the arytenoid cartilages almost entirely destroyed. The transverse diameter rima of the two lines; the inner wall of the trachla, from the point of division below, to the upper border of the cricoid cartilage is coated with a dense albuminous exudation, forming when detached, a perfect turbular membrane. The mucous membrane beneath of a deep purple color, readily detached from the elastic fibres beneath, resembling much in its general appearance a portion of stranglated intestine. Permission having been given only to examine the throat only, our investigation here ceased. Present at the examination besides myself Dr. Lewis and Dr. Pease.

